# On the March

**Published:** 1890-07-19

**Authors:** 


### The Doctor's Pulpit.

### ON THE MARCH.

This page of The Hospital has for the most part been devoted to questions dealing with the physical and mental well-being of readers themselves. Perhaps those readers will be not unwilling to forget themselves for a while and to go with us to those distant countries where our red coats uphold the honour of the British flag under circumstances not only of danger but sometimes of much distress and pain. No doubt we are all fighting apretty stiff daily battle of our own; and those who are in the conflict are apt to think they may be excused if they forget those who are far away. But we must not permit ourselves to forget that we are parts of a great nation, and that the nation has its conflict as well as the individual. Every red coat represents several hundred black coats, and quite as many frocks and bonnets as well. To have no regard for our soldiers is to have no regard for the national honour; and he who has no regard for the national honour deserves to both live and die in a ditch.

This preliminary is by way of introducing to readers of The Hospital a new waggon tent devised for armies on the march, and which is not only a tent, but a travelling hospital, a cooking kitchen, a drawing-room, a bedroom, and a defence against the enemy all in one. Do not throw the paper down, reader, and ask what you have to do with travelling tents and hospitals? Perhaps very little! But your neighbour may possibly have a son or a brother among the red-coats in India, or at the Cape, or even in some place still less contiguous to the comforts of home and civilization. For your neighbour's or your friend's sake, then, give a little thought to our soldiers; and still more for the sake of the soldiers themselves. Perhaps you are not old enough to remember the Crimean War! But some of us are; and we never read of the terrible and, in most cases, unnecessary sufferings of our brave soldiers in the trenches there without feeling a savage desire that two or three hundred army contractors and commissariat managers might be brought before a court martial and instantly shot "to encourage the others." We owe an incalculable debt to our armies, not only for the services they are constantly rendering us, but also by way of atonement for our shameful and unpardonable treatment of past generations of soldiers.

Some little time ago a meeting was held at the Royal United Service Institution, presided over by Director-General Sir T. Crawford, K.C.B. The object of the meeting was to consider the best method of transporting the sick and wounded in time of war. Surgeon William A. Morris, of the Army Medical Staff, described to the meeting a combined waggon and tent invented by Captain Tomkins, late of the Victoria Rifles. The waggon and tent together go by the appropriate name of the "Tortoise," probably because that animal, like the soldier on the march, must always carry his house about with him wherever he goes. The tent is designed to give to the wounded soldier all the advantages he would have if he could be suddenly transported by a feat of legerdemain to one of the best London hospitals. The waggon portion is so constructed as to be an ambulance waggon when moving, and to form a part of a large tent thirty feet long by twenty feet broad when at rest and in position. It carries within its ample space all the ordinary stores of a hospital tent, including stretcher beds, blankets, medicine chests, and three days' provisions for twenty patients. The tent pure and simple weighs only 368 pounds, and is made in four pieces, so that it can, when removed, be carried by two pack animals, or when these are not available by men. Notwithstanding its very small weight the tent is double throughout, and there is a large air space between the inner and outer canvass coverings. This, of course, makes it much warmer, and practically does away with those hateful attendants of tent life—draughts. The walls are made in sections and laced at the joints, so that the whole wall or any portion of it can be rolled or hitched up, or propped up like a verandah. A marked improvement is the presence of windows in the tent wall, which give light sufficient for surgical operations, and make the interior bright and cheerful. When the tent is not pitched the canvass forms a cover for the waggon; when it is pitched, the waggon forms a firm support and an interior apartment of the tent. So soon as an army stops on its march the "Tortoise" waggon can be converted into a pitched tent in fifteen minutes; and when the order is given for marching again, the tent can be struck and the waggon made ready for its journey also in fifteen minutes. Besides these advantages enumerated, the tent is so shaped as to resist wind pressure; its low pitch of roof and dark colour make it less visible to the enemy; the fact that the canvass is double permits lights to be used inside without revealing their presence outside; the tent being spread round the waggon enables the stores to be loaded and unloaded under cover; and the whole arrangement, waggon and tent included, and supplied with three days' provisions, eighteen stretcher beds, and cooking apparatus for fifty men, only weighs 4,600 pounds. It can thus easily be drawn by three horses, and even by two where roads are available. Moreover, the cooking stove can be used on the line of march, thus enabling the attendants to provide beef-tea, soup, hot water, and other comforts at any hour of the day or night.

There are two things to be said from a civilian point of view about Captain Tomkins' altogether admirable invention. The first is, that it is a deep satisfaction to the minds of all soldiers' relatives and of every other well-conditioned Britain to know that the soldier who is shot or sabred or speared can be instantly taken to a place of safety where surgical skill and ample appliances will be used for his comfort and for the saving of his life. The second is, that the invention having been brought into public notice, the public in turn must insist upon its being brought into immediate and general use. We are aware, of course, that economy and red tape are a very formidable pair of opponents. But economy of money is a monstrous crime when it results in waste of life. The man of sound sense and sober patriotism will insist that our wounded soldiers must have everything of the very best, whatever may be the cost. As for red tape, our patience with it is very limited. Public opinion is happily growing stronger day by day; and it will soon resolve that every permanent official in the War Department shall be swept into oblivion rather than that the brave soldiers, for whom alone the department exists, shall suffer a single hours' unnecessary pain and danger. We make no apology either for bringing this subject before our readers, or for the use of language which may be considered strong. Our soldiers and sailors are the custodians of the nation's honour, the guardians of her flag, and the defenders of her homes. Nothing that we can do for them can by any possibility be either too costly or too great. We have large arrears to make up, both in the way of payment of wages, and in the matter of kind treatment and honourable regard. That it should have been left to the private enterprise of a newspaper, the *St. James's Gazette*, to rescue some of the heroic "six hundred" from want, and not a few even from the workhouse, is a disgraceful fact in the history of our country. We are, as a race, both capable and proud. But we are not half so capable and proud as we ought to be. We should feel as much ashamed of the workhouse life of even one of the "six hundred" as a peer of the realm would if one of his own sons were in the workhouse. However much a nation may flatter itself on the strength of the glorious enterprises of its children, it cannot be a truly great nation unless its deeds, as a whole, are such as to commend themselves to the deepest instincts of generosity and justice. Shame, and not honour, will be the characteristic of our country if it does not give its fighting defenders a generous share of all that is best in its civilisation and its untold wealth.

